# Resequencing and transcriptomic analysis reveal differences in nitrite reductase in jujube fruit (*Ziziphus jujuba* Mill.)

**DOI:** 10.1186/s13007-021-00776-9

**Published:** 2021-07-12

**Authors:** Na Li, Yuqin Song, Jie Li, Ruijie Hao, Xinxin Feng, Liulin Li

**Affiliations:** 1 College of Horticulture, Shanxi Agricultural University, Taigu, 030801 China; 2 College of Forestry, Shanxi Agricultural University, Taigu, 030801 China

**Keywords:** *Ziziphus jujuba*, Transcriptome, Genome resequencing, InDel, Nitrite reductase

## Abstract

**Background:**

Jujube is a typical fruit tree species from China. ‘Muzao’, a cracking-susceptible cultivar, and ‘Linhuang No. 1’, a cracking-resistant cultivar, were selected in a previous study as contrasting research materials. Whole-genome resequencing and transcriptomic analysis of ‘Linhuang No. 1’ and ‘Muzao’ allowed the identification of differentially expressed genes with different gene structures between the two cultivars and could be helpful in explaining the differences and similarities between the two cultivars.

**Results:**

Resequencing identified 664,129 polymorphic variable sites between ‘Linhuang No. 1’ and ‘Muzao’. To determine the genetic relationship among ‘Linhuang No. 1’, ‘Muzao’ and the jujube genome reference cultivar ‘Dongzao’, the characteristic polymorphic variable sites were analysed by principal component analysis. The genetic relationship between ‘Linhuang No. 1’ and ‘Muzao’ was closer than that of either variety and ‘Dongzao’. Nineteen differentially expressed genes were identified by combining transcriptomic analysis with resequencing analysis. LOC107427052 (encoding a nitrite reductase) was identified by Kyoto Encyclopedia of Genes and Genomes (KEGG) enrichment analysis for further study. The identified insertion was not in the domain region of the LOC107427052 gene coding sequence (CDS) region and was verified by the finding that the insertion did not affect translation of the protein. The LOC107427052 gene expression levels, nitrite reductase activities and nitrite contents of ‘Muzao’ were significantly higher than the corresponding values of ‘Linhuang No. 1’ at the young fruit stage. There was no significant difference in the quantity of the product of nitrite reductase, namely, ammonia, between the two cultivars.

**Conclusions:**

The present study was the first to explore the differences between different jujube cultivars (‘Linhuang No. 1’ and ‘Muzao’) by combining genome resequencing and transcriptomics. LOC107427052 (encoding a nitrite reductase) was characterized by KEGG enrichment analysis. The insertion in the CDS region of the LOC107427052 gene provides a new direction for the study of nitrogen metabolism in jujube. Our study has laid a foundation for the comparative analysis of nitrite metabolism between the jujube cultivars ‘Linhuang No. 1’ and ‘Muzao’.

**Supplementary Information:**

The online version contains supplementary material available at 10.1186/s13007-021-00776-9.

## Introduction

Jujube is a typical fruit tree species from China [1]. Jujube is valued as a traditional herbal medicine as well as a popular fruit, eaten fresh or dried, and is cultivated on 2 million hectares in China alone, with an annual production of approximately 7.36 million tons in 2018 (https://data.stats.gov.cn/easyquery.htm?cn=E0103). Jujube cv. ‘Muzao’ is one of the most widely cultivated jujube varieties in China because of its high yield and high quality [2]. Due to the wide cultivation area of the jujube tree crop (*Ziziphus jujuba* Mill.), there are many varieties and geographical types of jujube, each varying in terms of fruit shape, individual fruit weight, drying rate, nutrient composition and fruit characteristics [3]. ‘Linhuang No. 1’ is a dry jujube cultivar selected from ‘Muzao’ that has fruit characteristics similar to those of ‘Muzao’. Compared with ‘Muzao’, however, ‘Linhuang No. 1’ shows greater resistance to fruit cracking [4]. ‘Linhuang No. 1’ has become a valuable genotype for scientific research into the mechanism of fruit cracking. Our previous study found that, in addition to the wax layer on the jujube fruit surface [5], changes in cell wall characteristics during ripening also played a very important role in jujube fruit cracking [6]. Changes in the composition and structure of the cell wall affect the mechanical strength of the cell wall and its associated tissues. However, the specific molecules involved in the mechanism of fruit cracking need to be further explored [6].

Whole-genome resequencing is the process of sequencing the genomes of different genotypes of a species where a draft genome has already been sequenced [7]. Through sequence alignment, this methodology can identify large numbers of single nucleotide polymorphisms (SNPs), insertion/deletion (InDel) variants, structural gene variants (SVs), copy number variants (CNVs) and other mutations in whole-genome resequenced individuals. This information on genetic variation is an important way to understand the genetic background of a species and to study its evolution. To explore the wide phenotypic diversity of sweet cherry varieties with respect to important agronomic traits, such as flowering time and defence response to pathogens, Aliki et al. [8] resequenced 21 cherry accessions and found that the majority of high-impact SNPs (e.g., addition of stop codons, introduction of frameshifts) were identified in genes involved in flowering time, dormancy and defence reactions against pathogens. Yu et al. [9] resequenced the genomes of 58 peach cultivars and closely related species to explore the origin and evolutionary history of peaches. The results showed that peaches originated on the Qinghai-Tibet Plateau in Southwest China and had been subjected to frugivore-mediated selection to drive the evolution of fruit traits. The publication of the ‘Dongzao’ genome sequence provided a valuable resource for genomic and transcriptomic studies of other jujube cultivars [10], not only for biological discovery and crop improvement but also for evolutionary and comparative genomic analysis [10]. Huang et al. [11] resequenced the genomes of 31 cultivated and wild jujube accessions to reveal the domestication process of jujube and identified key genes for metabolic effects associated with sweet or sour fruit. Guo et al. [12] resequenced the genome of 350 jujube accessions, including wild, semiwild and cultivated plants, to identify several candidate genes possibly involved in regulating seven domestication-related traits.

In this article, we present analyses of whole-genome resequencing and transcriptomics of ‘Linhuang No. 1’ and ‘Muzao’. The resequencing analysis focused on genomic regions associated with favourable variations, such as SNPs and InDels. The transcriptomics analysis focused on genes that were differentially expressed during the full-red period of fruit development. The combination of whole-genome resequencing and transcriptomics analysis allowed the identification of differentially expressed genes with different gene structures between ‘Linhuang No. 1’ and ‘Muzao’, which could be helpful in explaining elements of divergence and/or similarity with respect to cracking resistance between the two cultivars.

## Results

### Discovery of SNPs and InDels in ‘Linhuang No. 1’ and ‘Muzao’

A total of 110 million raw reads were generated for ‘Linhuang No. 1’ and ‘Muzao’. After the removal of low-quality reads, approximately 95.93% (‘Linhuang No. 1’) and 94.74% (‘Muzao’) of the reads were retained as clean data and used for further investigation. The Q20 percentage (proportion of nucleotides with a quality value greater than 20 within the read) was greater than 95%, and the Q30 percentage was greater than 89%. The mean GC percentage of the clean reads was 33%. The high-quality reads were further mapped to the reference genome (*Ziziphus jujuba* 1.1) using Burrows–Wheeler Aligner (BWA) software [13]. Overall, almost 94% of these reads were uniquely mapped and covered approximately 46.5% of the reference genome, with at least 30× coverage depth (Additional file 1: Table S1).

Compared with the *Z. jujuba* reference (‘Dongzao’) genome sequence, a total of 4,404,985 polymorphic sites, including 3,623,127 SNPs and 781,858 InDels, were discovered in ‘Linhuang No. 1’, with a total of 4,401,491 polymorphic sites, including 3,626,419 SNPs and 775,072 InDels, discovered in ‘Muzao’. From the comparison between ‘Linhuang No. 1’ and ‘Muzao’, 664,129 polymorphisms were detected. Table 1A summarizes the number of SNP and InDel variants relative to the reference genome sequence based on the individual chromosomes, whereas Table 1B details the number of polymorphisms between ‘Linhuang No. 1’ and ‘Muzao’ by chromosome. The highest number of SNPs and InDels between ‘Linhuang No. 1’ and the reference genome was detected for NC_029679.1 (Chromosome 1), whereas the lowest number of SNPs and InDels was detected for NC_029690.1 (Chromosome 12). For all chromosomes, InDel polymorphisms were less frequent than SNPs. From the comparison between ‘Muzao’ and the reference genome, the highest and lowest numbers of SNPs and InDels were also detected on chromosomes 1 and 12, respectively. From the comparison between ‘Linhuang No. 1’ and ‘Muzao’, the highest and lowest numbers of SNP and InDel polymorphisms were also found on chromosomes 1 and 12, respectively.

**Table 1:** Number of SNPs and InDels, according to chromosome (Ch), detected for ‘Linhuang No. 1’ and ‘Muzao’, relative to the reference genome ‘Dongzao’ (A), and the comparison between them (B)

A					B						
Chr	‘Linhuang No.1’		‘Muzao’		‘Linhuang No.1’ vs. ‘Muzao’						
	SNP	InDel	SNP	InDel	SNP	InDel					
						≤ 10 bp	11–20 bp	21–40 bp	41–80 bp	81–160 bp	≥ 161 bp
NC_029679.1	429,504	89,039	430,221	88,650	47,822	12,968	1437	930	780	310	43
NC_029680.1	246,814	54,159	246,954	53,764	30,426	8489	877	567	466	218	30
NC_029681.1	245,843	54,834	245,635	54,282	29,879	8761	921	553	495	174	26
NC_029682.1	228,631	48,207	229,082	47,855	32,212	8774	899	574	446	199	31
NC_029683.1	343,538	69,172	344,093	68,807	45,243	12,055	1303	776	615	222	40
NC_029684.1	217,230	47,474	217,519	46,967	25,026	7109	812	510	409	167	31
NC_029685.1	251,890	52,302	252,798	52,148	37,682	9423	991	659	510	185	39
NC_029686.1	182,760	42,746	182,578	42,337	22,962	6699	688	445	389	169	29
NC_029687.1	240,326	55,181	239,869	54,513	29,621	8750	814	540	461	171	36
NC_029688.1	195,933	41,950	196,006	41,515	22,887	6562	733	471	358	149	28
NC_029689.1	189,397	43,820	189,007	43,509	24,530	6804	776	468	322	129	22
NC_029690.1	155,137	35,962	155,452	35,687	18,755	5599	630	393	287	137	29
Unplaced genomic scaffold	696,124	147,012	697,205	145,038	127,817	34,303	3223	1936	1323	510	60
Total	3,623,127	781,858	3,626,419	775,072	494,862	136,296	14,104	8822	6861	2740	444

From the comparison between ‘Linhuang No. 1’ and ‘Muzao’ and the reference genome ‘Dongzao’, the highest and lowest numbers of SVs were also detected on chromosomes 1 and 12, respectively (Additional file 2: Table S2). From the comparison between ‘Linhuang No. 1’ and the reference genome, the highest and lowest numbers of CNVs were detected on NC_029682.1 (chromosome 4) and NC_029686.1 (chromosome 8), respectively. From the comparison between ‘Muzao’ and the reference genome, the highest and lowest numbers of CNVs were detected on NC_029683.1 (chromosome 5) and NC_029689.1 (chromosome 11), respectively (Additional file 2: Table S2).

**Fig. 1:**
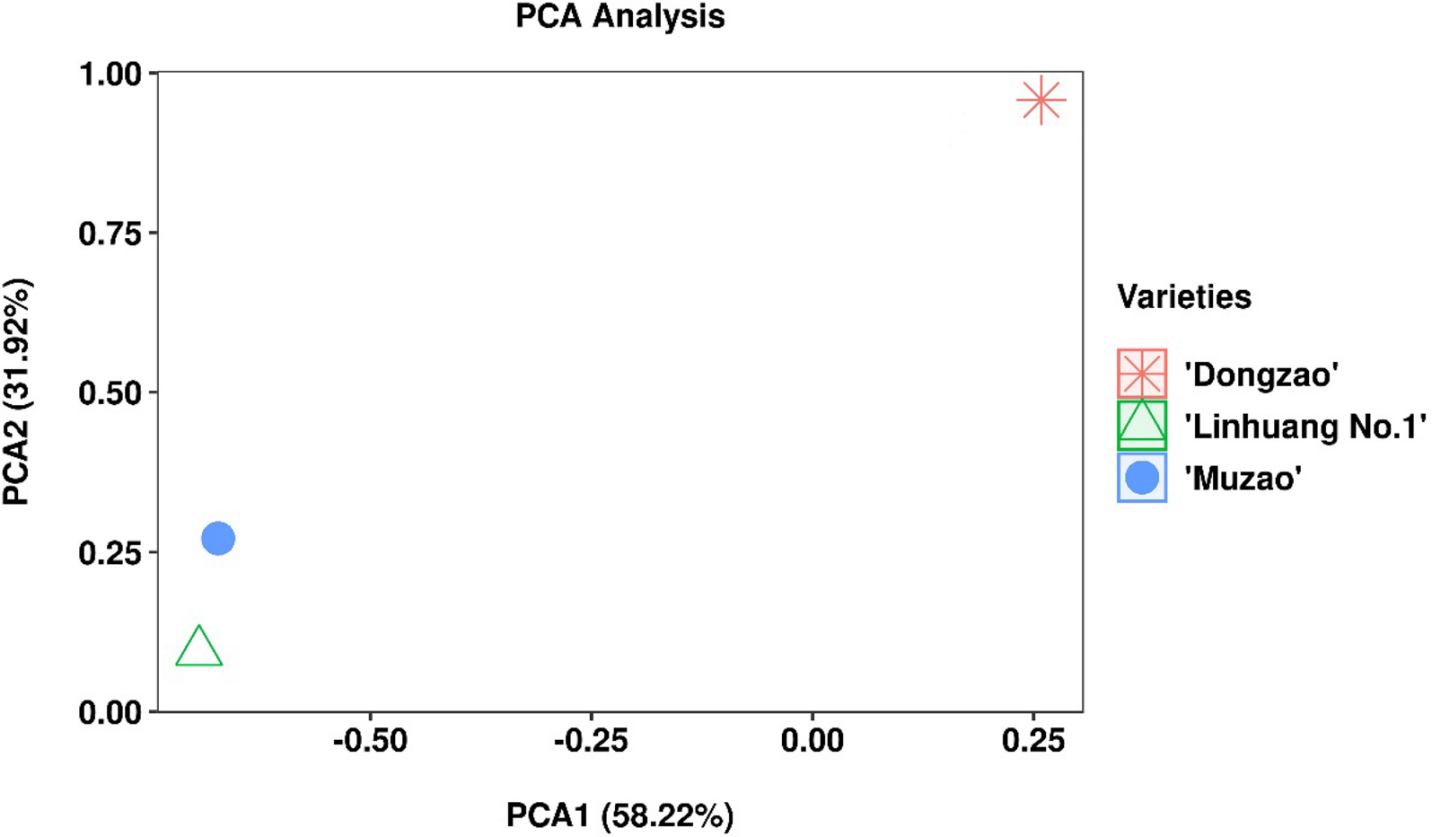
Principal component analysis (PCA) plot obtained from a similarity matrix among the *Ziziphus jujuba* Mill. cultivars ‘Linhuang No. 1’ and ‘Muzao’, relative to the jujube genome reference cultivar ‘Dongzao’ (ZizJuj_1.1, ID: 15586)

Figure 1 shows the relatedness among ‘Linhuang No. 1’, ‘Muzao’ and ‘Dongzao’. The distance between the points represents the degree of characteristic differences among the cultivars. The first and second principal components (PC1 and PC2) explained 58.22% and 31.92% of the total variation, respectively. The cumulative contribution rate of the two principal components was 90.14%. PC1 differentiated ‘Linhuang No. 1’ and ‘Muzao’ from ‘Dongzao’, whereas PC2 differentiated ‘Linhuang No. 1’, ‘Muzao’ and ‘Dongzao’. This confirmed that there were 664,129 polymorphisms between ‘Linhuang No. 1’ and ‘Muzao’.

**Fig. 2:**
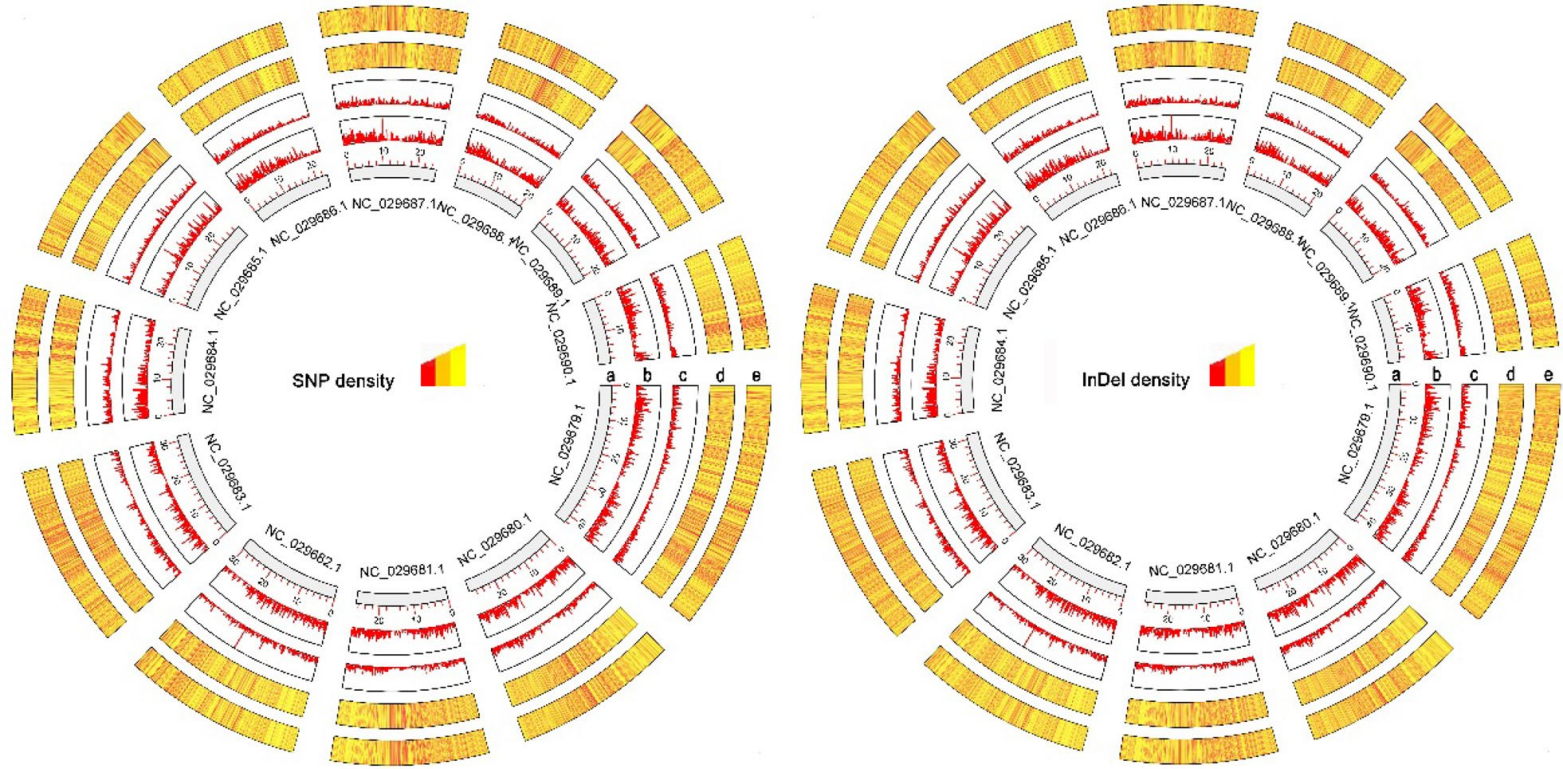
Single nucleotide polymorphism (SNP) and insertion/deletion (InDel) density plots by chromosome for ‘Linhuang No. 1’ and ‘Muzao’ against the reference genome ‘Dongzao’. **a** Chromosome scale; **b** gene density of the positive strand; **c** gene density of the negative strand; **d** ‘Linhuang No. 1’; e: ‘Muzao’

SNP and InDel density plots obtained through the comparison of ‘Linhuang No. 1’ and ‘Muzao’ against the reference genome ‘Dongzao’ are displayed in Fig. 2. This figure also provides an integrated view of the polymorphisms detected across the entire genome between ‘Linhuang No. 1’ and ‘Muzao’ in relation to the reference genome. ‘Linhuang No. 1’ has a similar level of polymorphism as ‘Muzao’ when compared with the reference genome.

### Differential expression of different structural genes between ‘Linhuang No. 1’ and ‘Muzao’

**Fig. 3:**
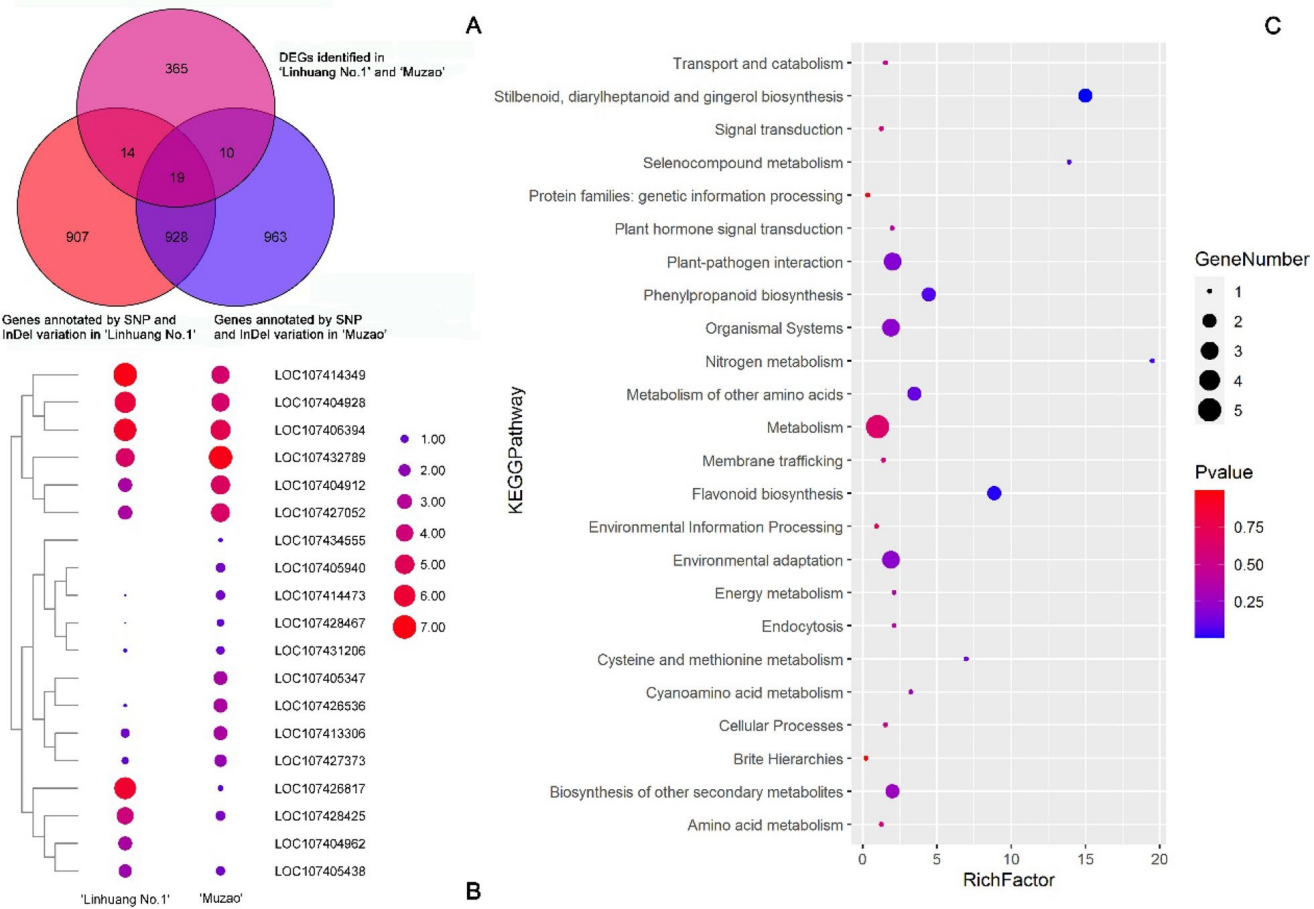
Venn diagram of 19 differentially expressed genes (DEGs) and genes containing single nucleotide polymorphisms (SNPs) and insertions/deletions (InDels). **A** Venn diagram of DEGs and SNP and InDel variants in ‘Linhuang No. 1’ and ‘Muzao’; **B** differential expression heat map of 19 DEGs; **C** enriched Kyoto Encyclopedia of Genes and Genomes (KEGG) pathways of 19 genes

To further explore the differentially expressed genes between ‘Linhuang No. 1’ and ‘Muzao’, combined resequencing and transcriptome analysis (SRP307646) of ‘Linhuang No. 1’ and ‘Muzao’ was carried out. The results showed that there were 33 SNP and 29 InDel variants in the differentially expressed genes between ‘Linhuang No. 1’ and ‘Muzao’. A total of 947 genes in ‘Linhuang No. 1’ and ‘Muzao’ contained SNPs or InDels, and 19 genes were differentially expressed between the two cultivars (Fig. 3A).

We performed cluster analysis on the 19 DEGs (Fig. 3B). We analysed clustering of the log2 FPKM (Fragments Per Kilobase of transcript per Million mapped reads) value, representing the gene expression. A colour closer to red represents higher gene expression. Of the 19 differentially expressed genes (DEGs), 12 genes were upregulated in ‘Muzao’: LOC107413306, LOC107427052, LOC107431206, LOC107432789, LOC107427373, LOC107404912, LOC107428467, LOC107426536, LOC107414473, LOC107434555, LOC107405940, and LOC107405347.

The 19 DEGs were further examined by Kyoto Encyclopedia of Genes and Genomes (KEGG) enrichment analysis (Fig. 3C, Table 2). According to the standard significance of *p* < 0.05, there were three enriched pathways in ‘Linhuang No. 1’ and ‘Muzao’, namely, stilbenoid, diarylheptanoid and gingerol biosynthesis (rich factor: 14.98), flavonoid biosynthesis (rich factor: 8.85) and nitrogen metabolism (rich factor: 19.48). Nitrogen metabolism (LOC107427052) was selected to further verify the transcriptomic data.

**Table 2:** Enriched pathways of 19 differentially expressed genes (DEGs)

	Pathway ID	Pathway	*p*-value	Genes
1	ko00945	Stilbenoid, diarylheptanoid and gingerol biosynthesis	0.00697	LOC107428425, LOC107428467
2	ko00941	Flavonoid biosynthesis	0.01965	LOC107428425, LOC107428467
3	ko00910	Nitrogen metabolism	0.04939	LOC107427052
4	ko00450	Selenocompound metabolism	0.06990	LOC107404928
5	ko00940	Phenylpropanoid biosynthesis	0.07128	LOC107428425, LOC107428467
6	ko09106	Metabolism of other amino acids	0.10862	LOC107404928, LOC107414349
7	ko00270	Cysteine and methionine metabolism	0.13537	LOC107404928
8	ko04626	Plant-pathogen interaction	0.17866	LOC107414473, LOC107434555, LOC107413306
9	ko09159	Environmental adaptation	0.20606	LOC107414473, LOC107434555, LOC107413306
10	ko09150	Organismal systems	0.20606	LOC107414473, LOC107434555, LOC107413306
11	ko09110	Biosynthesis of other secondary metabolites	0.26609	LOC107428425, LOC107428467
12	ko00460	Cyanoamino acid metabolism	0.26972	LOC107414349
13	ko04144	Endocytosis	0.38498	LOC107404962
14	ko09102	Energy metabolism	0.38498	LOC107427052
15	ko04075	Plant hormone signal transduction	0.40467	LOC107432789
16	ko09140	Cellular processes	0.49486	LOC107404962]
17	ko09141	Transport and catabolism	0.49486	LOC107404962
18	ko04131	Membrane trafficking	0.52736	LOC107404962
19	ko09132	Signal transduction	0.56775	LOC107432789
20	ko09105	Amino acid metabolism	0.56775	LOC107404928
21	ko09100	Metabolism	0.63880	LOC107428425, LOC107428467, LOC107427052, LOC107404928, LOC107414349
22	ko09130	Environmental information processing	0.67476	LOC107432789
23	ko09182	Protein families: genetic information processing	0.97181	LOC107404962
24	ko09180	Brite hierarchies	0.99815	LOC107404962

### Levels of nitrite reductase (NiR) activity and nitrite and ammonia nitrogen concentrations

**Fig. 4:**
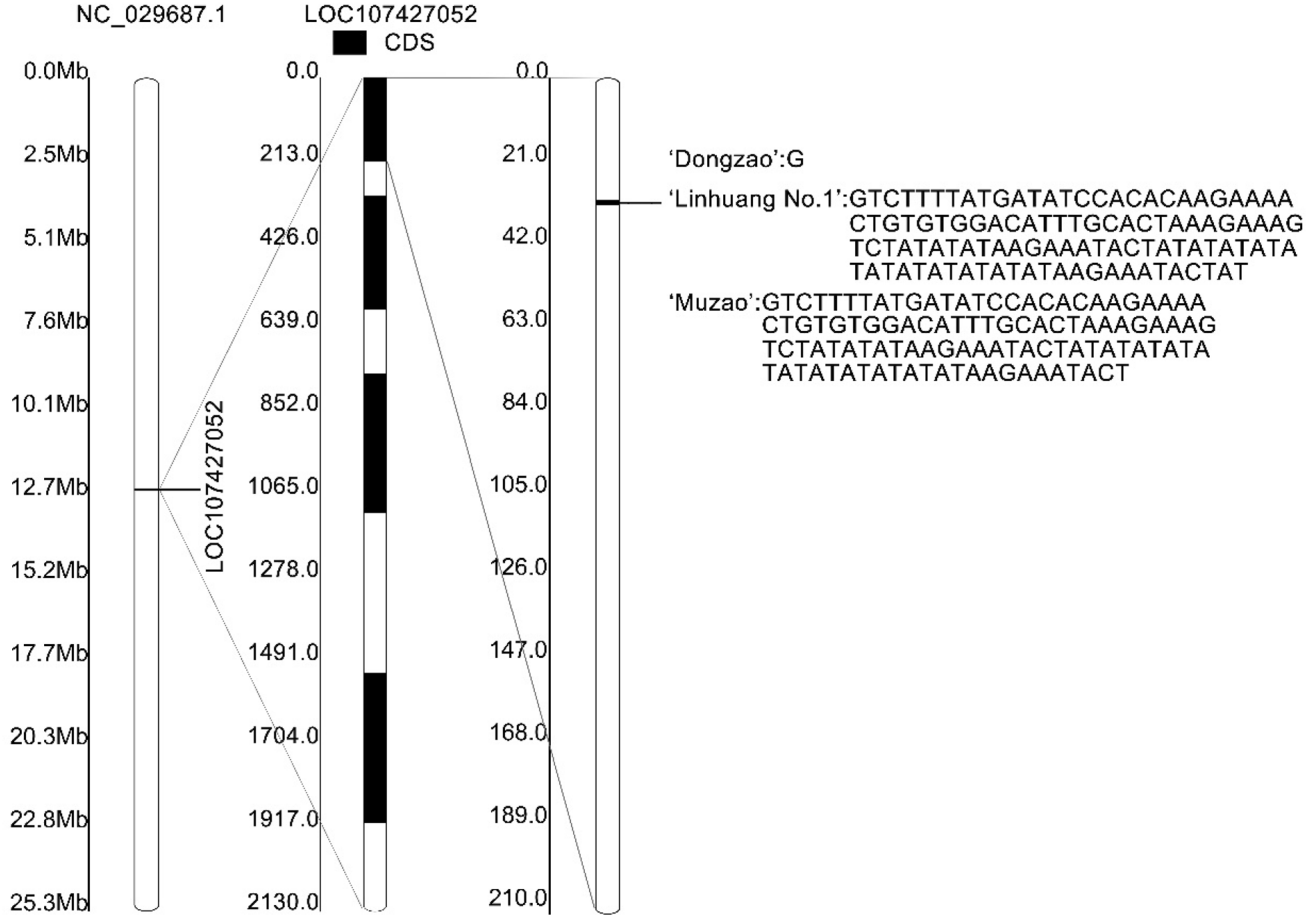
LOC107427052 polymorphism in ‘Linhuang No. 1’ and ‘Muzao’

**Fig. 5:**
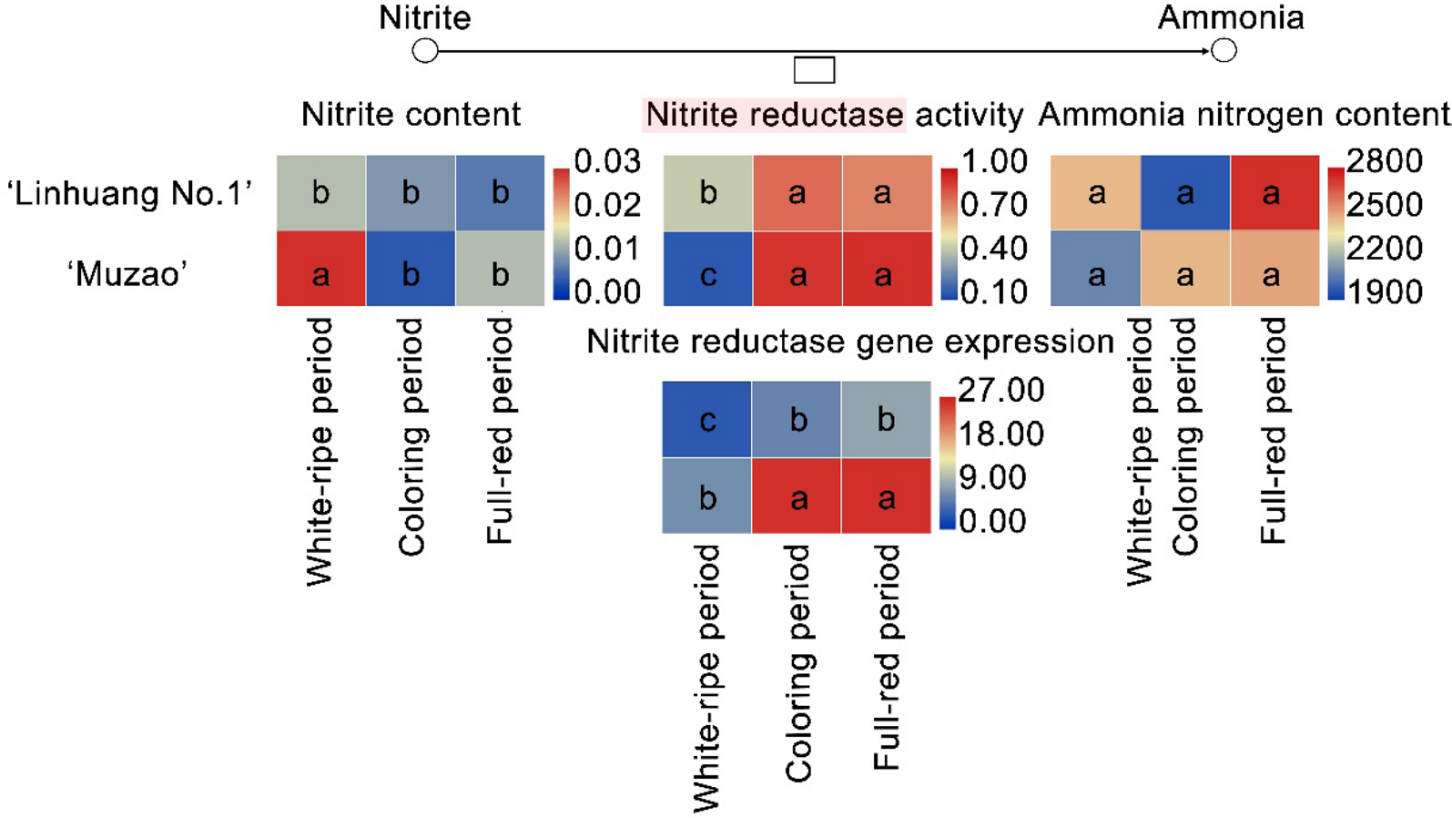
Levels of nitrite reductase (NiR) activity and gene expression and concentrations of nitrite and ammonia nitrogen at different stages of fruit development in the two cultivars. For a particular variable, samples with the same letter are not significantly different (*p* > 0.05)

LOC107427052 encodes a nitrite reductase. In the process of assimilating nitrate nitrogen in plants, nitrite reductase (NiR) is coupled with nitrate reductase (NR) to complete the inorganic assimilation of nitrate. Nitrite reductase can catalytically reduce nitrite to ammonia. Nitrite reductase plays a very important role in the growth and development of plants and is widespread in the leaves and roots of higher plants. Based on the resequencing results of ‘Linhuang No. 1’ and ‘Muzao’, InDel events occurred in the LOC107427052 sequences of both varieties compared with ‘Dongzao’ (Fig. 4). A total of 106 bases were inserted at position 12,517,784 (in the coding sequence (CDS) region) on chromosome NC_029687.1 of ‘Linhuang No. 1’, while 104 bases were inserted at the same position in ‘Muzao’. To confirm the resequencing results, the LOC107427052 gene was cloned using cDNA from ‘Linhuang No. 1’ and ‘Muzao’. The protein sequences encoded by the LOC107427052 genes from ‘Dongzao’, ‘Linhuang No. 1’ and ‘Muzao’ were compared, and their similarity was 91.82% (Additional file 3: Figure S1, Additional file 4). Domain prediction was performed on the protein sequence, and it was found that the insertion in ‘Linhuang No. 1’ and ‘Muzao’ did not occur in the domain region of the enzyme (Additional file 5: Figure S2). Real-time quantitative PCR (qPCR) was used to verify the expression level of the LOC107427052 gene (Fig. 5). The results showed that the LOC107427052 gene expression levels of ‘Muzao’ were significantly higher than those of ‘Linhuang No. 1’ during fruit development and that the expression levels of the LOC107427052 gene in ‘Linhuang No. 1’ and ‘Muzao’ increased gradually as the fruit developed. The expression level of the LOC107427052 gene was significantly higher during the colouring period and the full-red period than during the young fruit period.

Additionally, the nitrite reductase activities of ‘Linhuang No. 1’ and ‘Muzao’ were determined at different fruit developmental stages. The results showed that there was no significant difference in the nitrite reductase activities between ‘Linhuang No. 1’ and ‘Muzao’ at the later stage of fruit development; the nitrite reductase activities of ‘Linhuang No. 1’ and ‘Muzao’ were 0.75 U/g fresh weight (FW) and 0.93 U/g FW (*p* < 0.05) at the full-red stage, respectively. However, the nitrite reductase activities of ‘Muzao’ were significantly higher than those of ‘Linhuang No. 1’ at the young fruit stage (*p* > 0.05). The nitrite content of ‘Muzao’ at the young fruit stage was significantly higher than that of ‘Linhuang No. 1’ (*p* > 0.05). There was no significant difference in the concentration of the NiR product ammonia between the two varieties at the young fruit stage.

## Discussion

The Chinese jujube tree was domesticated from the wild jujube (*Z. jujuba* Mill. var. *spinosa* Hu.) [14]. Cultivated jujube trees and wild jujube show different characteristics, such as tree vs. shrub habits, sparsely thorned vs. heavily thorned stems, and large vs. small fruits, respectively, due to artificial selection for important agronomic characters [10, 12, 15]. Most cultivated jujube varieties produce relatively fewer seeds due to self-incompatibility or cross-incompatibility. Jujube trees can only be improved through seed selection, selection of bud mutations or molecular breeding [16]. These genetic methods have resulted in the high heterozygosity, high repeat sequence density and low GC content of the jujube genome [16]. Therefore, sequencing of the jujube genome has been very difficult. Liu et al. [10] published the genome of the first cultivar of the genus, *Ziziphus jujube* cv. ‘Dongzao’ (437.65 Mbp). The release of this genome sequence provided a rich resource of genetic information for the breeding of improved jujube accessions, as well as for the molecular improvement of other plants and fruit trees in the family Rhamnaceae.

Huang et al. [11] reported the genome sequence of another jujube variety, ‘Junzao’, and resequenced the genomes of 31 wild jujube and domesticated jujube accessions with different geographical distributions. Huang and colleagues revealed the genomic mechanism underlying the improvement of fruit sweetness and acidity during domestication and identified four genes related to acidic metabolism pathways; these genes encode an NADP-dependent malic enzyme, a pyruvate kinase, an isocitrate dehydrogenase and an aconitate hydratase, which play key roles in organic acid metabolism. This research has provided valuable genomic information and improved material for jujube breeding. Guo et al. [12] reported the resequencing of 350 jujube accessions. Through a genome-wide association study (GWAS) and selective sweep analysis, variation in the genes involved in the regulation of seven domestication traits was identified, including fruit shape, kernel size and fruiting branch length. This study provided rich genomic resources for revealing the genetic basis of the domestication and evolution of jujube.

In the current study, the varieties ‘Linhuang No. 1’ (resistant to cracking) and ‘Muzao’ (susceptible to cracking) were used as the research materials, with ‘Dongzao’ used as the reference genome for resequencing analysis. A total of 4,404,985 and 4,401,491 polymorphic sites were obtained in ‘Linhuang No. 1’ and ‘Muzao’, respectively, whereas 664,129 variant sites were found between ‘Linhuang No. 1’ and ‘Muzao’. Principal component analysis of the characteristic variant sites between ‘Linhuang No. 1’, ‘Muzao’ and ‘Dongzao’ was performed, and the results showed that both PC1 and PC2 could distinguish ‘Linhuang No. 1’ and ‘Muzao’ from ‘Dongzao’, indicating that ‘Linhuang No. 1’ and ‘Muzao’ were closer in relation to one another than to ‘Dongzao’.

Mutation is a key element in species evolution. Widespread base substitution and insertion/deletion mutations are important driving forces for genome evolution [17, 18]. Insertion and deletion mutations are more likely to trigger species evolution than are base substitution mutations. The greater the evolutionary distance between species, the more likely bases are inserted or deleted and the greater the length of the insertions or deletions [19–21]. Insertions and deletions are the main reasons for the divergence of closely related species. The insertion and deletion of bases can cause DNA sequence changes and DNA fragment length polymorphisms. They can even change the structure of genes through insertions or deletions in the exons and introns of the original gene, leading to the generation of new genes in the genome [22]. Resequencing revealed 781,858 and 775,072 insertions or deletions in ‘Linhuang No. 1’ and ‘Muzao’, respectively. Relative to ‘Muzao’, there were 169,267 insertions and deletions in ‘Linhuang No. 1’. To a large extent, these polymorphisms led to the different characteristics observed between ‘Linhuang No. 1’ and ‘Muzao’.

The transcriptome combines the genetic information of the genome with that of the proteome, including biological functions based on RNA, uses high-throughput sequencing technology (RNA-Seq) to sequence all cDNA libraries in tissues or cells and calculates the gene expression under different processing conditions (by counting the number of relevant reads). The jujube transcriptome has been widely used in research on the response to heat stress, cold stress, salt stress and colouring of jujube fruits [23–25]. In the current study, 431 differentially expressed genes were identified by transcriptome analysis of ‘Linhuang No. 1’ and ‘Muzao’ pericarps. At the same time, the transcriptome and resequencing analyses were combined to identify the differentially expressed genes in ‘Linhuang No. 1’ and ‘Muzao’ with respect to structural polymorphisms. A total of 19 mutant genes were identified as exhibiting differential expression. There were three significantly enriched pathways in ‘Linhuang No. 1’ and ‘Muzao’: stilbenoid, diarylheptanoid and gingerol biosynthesis (rich factor: 14.98), flavonoid biosynthesis (rich factor: 8.85) and nitrogen metabolism (rich factor: 19.48). The gene LOC107427052, encoding a nitrite reductase in the nitrogen metabolism pathway, was identified for further study.

There is a relationship between nitrogen metabolism and cell wall growth regulation. During nitrogen assimilation, disassembly of the cell wall could be necessary for enhanced nitrate uptake, allowing for adequate cell and plant growth. This balance of cell wall loosening and thickening could be enhanced by nitrogen assimilation [26]. The physical and chemical properties of the cell wall lead to differences in the mechanical properties of the jujube pericarp and affect susceptibility to fruit cracking [27]. Martin and Moritz showed that the cracking of sweet cherry fruit was related to epidermal cell wall characteristics, with the expansion of the cell wall affecting the breaking mode, firmness and pressure of the fruit pericarp [28].

In the process of nitrate assimilation in plants, nitrite reductase (NiR) is coupled with nitrate reductase (NR) to complete the inorganic assimilation of nitrate. Nitrite reductase can catalytically reduce nitrate to ammonium [29]. In fact, the reduction rate of nitrate must be strictly regulated. It is necessary to ensure that nitrite and ammonium will not be excessive to avoid plant poisoning, and a sufficient supply of ammonium must be ensured. In this process, NiR plays a role in connecting the past and the future. Sivasankar et al. found that the nitrate inducible element in a gene is located in the promoter between the upstream positions 230 and 180, while the downstream positions 1 to 67 are very important for the minimal induction of nitrate [30]. Ozawa and Kawahigashi cloned the NiR gene of rice cv. ‘Konansou’ and overexpressed the gene in a commercial rice cultivar, ‘Koshihikari’ [31]. The results showed that, compared with the wild-type ‘Koshihikari’, the introduced exogenous NiR gene made the transgenic plant grow better, while callus regeneration ability was also significantly improved [31].

Compared with the reference genome ‘Dongzao’, ‘Linhuang No. 1’ had an insertion of 106 bases at position 12,517,784 (CDS region) on chromosome NC_029681.1, whereas ‘Muzao’ had an insertion of 104 bases at the same position; both insertions were verified by cloning. Insertion/deletion mutations are rarer than base substitutions in the evolution of organisms. If the sequence of the coding region has an InDel mutation involving a base sequence that is not a multiple of three bases, it will cause a frameshift mutation with potentially serious consequences, which places the mutant under greater negative selection. In the present study, the domain of the NiR protein was predicted from the cloned sequence, and it was found that the insertion did not occur in the domain region. The expression levels of the LOC107427052 genes of ‘Linhuang No. 1’ and ‘Muzao’ were consistent with the changes found in the nitrite reductase activity during fruit development. However, the LOC107427052 gene expression level of ‘Muzao’ was significantly higher than that of ‘Linhuang No. 1’ during fruit development. The nitrite reductase activities and the nitrite content of ‘Muzao’ were significantly higher than those of ‘Linhuang No. 1’ at the young fruit stage. However, there was no significant difference in the concentration of the NiR product, ammonia, between the two cultivars.

## Conclusions

The present study was the first to explore the differences between different jujube cultivars (‘Linhuang No. 1’ and ‘Muzao’) by combining genome resequencing and transcriptomics. A total of 664,129 polymorphism variable sites were found between ‘Linhuang No. 1’ and ‘Muzao’. The characteristic polymorphic variable sites between ‘Linhuang No. 1’, ‘Muzao’ and ‘Dongzao’ were analysed by principal component analysis. The genetic relationship between ‘Linhuang No. 1’ and ‘Muzao’ was closer than those between either cultivar and ‘Dongzao’. A total of 431 differentially expressed genes were identified by transcriptomics, and 19 differentially expressed genes were identified by combining transcriptomics with resequencing analysis. LOC107427052 (encoding a nitrite reductase) was characterized by KEGG enrichment analysis for further study. There was a 106/104-base insertion in the CDS region of the LOC107427052 gene, which provides a new direction for the study of nitrogen metabolism in jujube. Our study has laid a foundation for the analysis of genetic information and comparative nitrogen metabolism between ‘Linhuang No. 1’ and ‘Muzao’.

## Materials and methods

### Plant materials

Fruits and leaves of ‘Linhuang No. 1’ and ‘Muzao’ were harvested from trees from the Jujube Germplasm Resource Nursery, Pomology Institute, Shanxi Agricultural University, Shanxi, China. The ‘Linhuang No. 1’ and ‘Muzao’ plants used for sampling were grafted onto the same tree. The trees were cultivated according to current regulations for integrated fruit production in this region. Fruits and leaves without disease or physical injuries were selected for sampling. All fruits and leaves were packed and delivered immediately to the laboratory at Shanxi Agricultural University.

### Genome re-sequencing

Young leaves of ‘Linhuang No. 1’ and ‘Muzao’ were collected and stored at − 80 °C prior to DNA isolation. Genomic DNA was extracted using a modified CTAB method [11]. The sequencing library (30×) was prepared according to the standard protocol of Illumina, and sequencing was conducted by LC bio, Hangzhou, China, using an Illumina NovaSeq 6000. Clean data were aligned to the jujube reference genome ‘Dongzao’ (https://ftp.ncbi.nlm.nih.gov/genomes/all/GCF/000/826/755/GCF_000826755.1_ZizJuj_1.1/GCF_000826755.1_ZizJuj_1.1_genomic.fna.gz) using Burrows–Wheeler Aligner (BWA) software [13]. Mark Duplicates in Picard (https://sourceforge.net/projects/picard/) was used to eliminate PCR duplications. We used the Genome Analysis Toolkit (GATK) for base recalibration and realignment near insertion or deletion regions [13]. SAMtools was used to estimate reference genome coverage of SNP and InDel variants for identification and analysis.

### Gene cloning and protein sequence alignment

The target gene encoding nitrite reductase (NiR) was amplified using cDNA from the pericarp of ‘Linhuang No. 1’ and ‘Muzao’ fruits. The primers used were forward primer ATGTCATCGTTCTCTGTTCGGTTT and reverse primer TCAAAACGGGTGTTTCCCTCGA. The amplified products were electrophoresed on a 1% agarose gel and photographed with a gel imaging analysis system. The PCR products were recovered and purified according to the instructions for the Gel Recovery Kit (Takara, Kyoto, Japan). The recovered DNA fragment was ligated with a TA cloning vector and transformed into *Escherichia coli* DH5α. After 12–16 h of inverted culture at 37 °C, single colonies were selected and cultured in 2 mL lysogeny broth (LB) liquid medium. After 5–6 h of shake culture (150 rpm) at 37 °C, PstI digestion was used for identification. The bacterial suspension was stored in a final concentration of approximately 25% (v/v) glycerin, and a 100-μL aliquot of the bacterial suspension was sent to Sangon Biotech (Shanghai, China) for sequencing. The cloned sequences were subjected to domain analysis by TBtools [32] and NCBI-CD Search (https://www.ncbi.nlm.nih.gov/Structure/cdd/wrpsb.cgi).

### Quantitative real-time PCR validation of nitrite reductase gene expression levels

Quantitative real-time PCR (qPCR) was performed using ChamQTM Universal SYBR qPCR Master Mix (Vazyme, Nanjing, China). The cDNA templates were reverse transcribed using total RNA extracted from the pericarp of fruits of ‘Linhuang No. 1’ and ‘Muzao’ harvested at different developmental periods. Then, the nitrite reductase gene (LOC107427052) was PCR amplified. The primers were forward GCAAATCCGTGGTGTGGTT and reverse CAGCAAGAGGGTTCCCAACT. The jujube histone (HIS) gene (GenBank accession No. EU916201) was used as the internal reference for the gene expression analysis. The real-time PCR assay mix (20 μL) consisted of 2 μL of cDNA sample (diluted 1:10), 10 μL of 2× ChamQ Universal SYBR qPCR Master Mix (Vazyme, Nanjing, China), 0.6 μL of each primer (10 µM) and 6.8 μL of distilled deionized H_2_O. Each PCR assay was run on an iCycler iQ Real-Time PCR Detection System (Bio-Rad, California, USA) using the method described in a previous study [5]. The 2^−ΔΔCq^ method was used to calculate the relative abundance of transcripts present in each PCR amplification mixture.

### Determination of nitrite reductase (NiR) activity and concentrations of nitrite and ammonia nitrogen

Determinations of the activity of nitrite reductase (NiR) and the concentrations of nitrite and ammonia nitrogen were carried out according to the Nitrite Reductase (NiR) Activity Assay Kit (BioBox, Beijing, China), Nitrite Content Assay Kit (BioBox, Beijing, China) and Plant Ammonia Nitrogen Content Assay Kit (BioBox, Beijing, China), respectively, following the manufacturer’s instructions. The pericarp of fruits harvested from ‘Linhuang No. 1’ and ‘Muzao’ at different stages of fruit development were taken as samples. Independent analysis of each cultivar × development stage combination was carried out three times.

### Data analysis

SAS System 8.6 (SAS Institute, Cary, NC, USA) was used to analyse the data. Data analysis was carried out by one-way analysis of variance (ANOVA), with Student’s t test being used to make multiple pairwise comparisons to determine the significant differences among samples. Differences with *p* < 0.05 were statistically significant. Figures were constructed using Excel 2016, TBtools and Origin 9.0 (Microcal Software Inc., Northampton, MA, USA).

## Supplementary Information

**Additional file 1: Table S1.** Summary of sequencing and mapping results.

**Additional file 2: Table S2.** Distribution of CNVs and SVs across the chromosomes.

**Additional file 3: Figure S1.** Sequence alignment of the ‘Dongzao’, ‘Linhuang No. 1’ and ‘Muzao’ LOC107427052 genes.

**Additional file 4.** LOC107427052 protein sequences from ‘Dongzao’, ‘Linhuang No. 1’ and ‘Muzao’.

**Additional file 5: Figure S2.** Domain prediction of the ‘Dongzao’, ‘Linhuang No. 1’ and ‘Muzao’ LOC107427052 genes.

## Data Availability

All data generated or analysed during this study are included in the main paper and supplementary information files. In addition, whole-genome resequencing and RNA-seq data in this study were uploaded to the National Center for Biotechnology Information (NCBI) Sequence Read Archive (SRA) database under accession numbers SRP307848 and SRP307646, respectively.

## Abbreviations

SNP: Single nucleotide polymorphism
InDel: Insertion/deletion variant
SV: Structural gene variant
CNV: Copy number variant
DEGs: Differentially expressed genes
KEGG: Kyoto Encyclopedia of Genes and Genomes
FPKM: Fragments Per Kilobase of transcript per Million mapped reads
NiR: Nitrite reductase
NR: Nitrate reductase
CDS: Coding sequence
