# Supplementary figures and images for: Resequencing and transcriptomic analysis reveal differences in nitrite reductase in jujube fruit (Ziziphus jujuba Mill.)
Source: Plant Methods. 2021 Jul 12;17:75. doi: 10.1186/s13007-021-00776-9 (PMC8274035; doi:10.1186/s13007-021-00776-9)

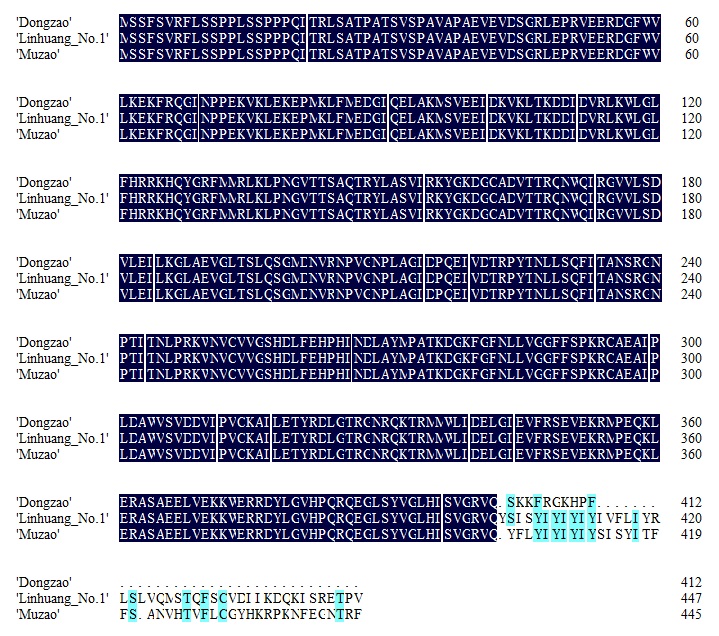

Supplement: Supplementary file 3 — Additional file 3: Figure S1. Sequence alignment of the ‘Dongzao’, ‘Linhuang No. 1’ and ‘Muzao’ LOC107427052 genes. [file 13007_2021_776_MOESM3_ESM.jpg]

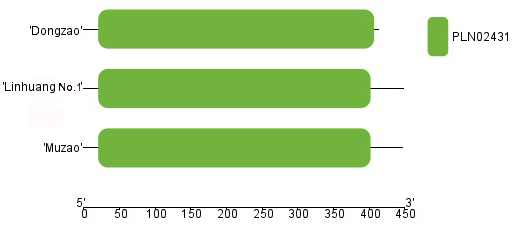

Supplement: Supplementary file 5 — Additional file 5: Figure S2. Domain prediction of the ‘Dongzao’, ‘Linhuang No. 1’ and ‘Muzao’ LOC107427052 genes. [file 13007_2021_776_MOESM5_ESM.jpg]
